# Radiomics based on multiparametric MRI for extrathyroidal extension feature prediction in papillary thyroid cancer

**DOI:** 10.1186/s12880-021-00553-z

**Published:** 2021-02-09

**Authors:** Ran Wei, Hao Wang, Lanyun Wang, Wenjuan Hu, Xilin Sun, Zedong Dai, Jie Zhu, Hong Li, Yaqiong Ge, Bin Song

**Affiliations:** 1 Department of Radiology, Minhang Hospital, Fudan University, 170 Xinsong Road, Shanghai, 201199 People’s Republic of China; 2 GE Healthcare, Shanghai, People’s Republic of China

**Keywords:** Radiomics, Papillary thyroid carcinoma, Extrathyroidal extension, Magnetic resonance imaging

## Abstract

**Background:**

To determine the predictive capability of MRI-based radiomics for extrathyroidal extension detection in papillary thyroid cancer (PTC) pre-surgically.

**Methods:**

The present retrospective trial assessed individuals with thyroid nodules examined by multiparametric MRI and subsequently administered thyroid surgery. Diagnosis and extrathyroidal extension (ETE) feature of PTC were based on pathological assessment. The thyroid tumors underwent manual segmentation, for radiomic feature extraction. Participants were randomized to the training and testing cohorts, at a ratio of 7:3. The mRMR (maximum correlation minimum redundancy) algorithm and the least absolute shrinkage and selection operator were utilized for radiomics feature selection. Then, a radiomics predictive model was generated via a linear combination of the features. The model’s performance in distinguishing the ETE feature of PTC was assessed by analyzing the receiver operating characteristic curve.

**Results:**

Totally 132 patients were assessed in this study, including 92 and 40 in the training and test cohorts, respectively). Next, the 16 top-performing features, including 4, 7 and 5 from diffusion weighted (DWI), T2-weighted (T2 WI), and contrast-enhanced T1-weighted (CE-T1WI) images, respectively, were finally retained to construct the radiomics signature. There were 8 RLM, 5 CM, 2 shape, and 1 SZM features. The radiomics prediction model achieved AUCs of 0.96 and 0.87 in the training and testing sets, respectively.

**Conclusions:**

Our study indicated that MRI radiomics approach had the potential to stratify patients based on ETE in PTCs preoperatively.

## Background

Papillary thyroid carcinoma (PTC) constitutes about 80% of all differentiated thyroid cancers, representing the commonest type of thyroid cancer [1]. PTC shows aggressive properties, including extrathyroidal extension (ETE), and lymph node and distant metastases, suggesting poor prognosis [2, 3]. ETE reflects a primary tumor that extends beyond the thyroid capsule and invades the neighboring tissues [4]; it is considered to have an elevated risk of local recurrence [5, 6] and utilized in multiple staging systems [7–9]. Based on the degree of invasion, ETE is divided into minimal and gross ETE. Traditional treatment options for PTC include total and subtotal thyroidectomies, with or without cervical lymph node dissection, and subsequent radioactive iodine remnant ablation [10]. However, PTC risk is relatively low, with recurrence and survival rates of 3–4% and > 99%, respectively [11]. According to the 2015 ATA Guidelines [12], ipsilateral lobectomy is recommended rather than total thyroidectomy in low risk patients with PTC, and thyroidectomy with prophylactic central cervical lymphadenectomy is not recommended for non-aggressive PTCs because of complications, including laryngeal nerve injury and hypoparathyroidism. Currently, the aggressive properties of tumors, especially the ETE feature, can only be obtained by pathological evaluation of specimens after thyroidectomy [13]. Therefore, preoperative assessment of PTC aggressiveness may help clinicians better plan surgical procedures. This suggests that noninvasive examination methods for identifying the aggressiveness of tumors are urgently needed for more targeted treatment.

Ultrasound (US) represents the commonest imaging method for thyroid nodule detection. However, its accuracy in assessing deep neck structures is not satisfactory due to the influence of bones and air [12, 14]. Furthermore, US is ambiguous for minor extrathyroidal extension [15, 16]. Fine-needle aspiration (FNA) biopsy can be accurately and cost-effectively applied [12], but has low ability in revealing the aggressive features of thyroid nodules [3, 17, 18].

Magnetic resonance imaging (MRI) can provide excellent contrast of soft tissues and allow multi-planar evaluation of anatomical details. MRI also assesses tumor aggressiveness, such as the ETE feature and cervical lymph node metastasis [19–21]. Another study [3] demonstrated that DW-MRI-based ADC values could help stratify PTC patients according to the ETE, although the average ADC of ROIs utilized might not comprehensively reflect the tumor features. Identification of an effective non-invasive imaging approach would provide insights into early PTC management.

Radiomics represents a high-throughput quantitative feature extraction method, which converts images into minable data, and then analyzes these data to provide decision support. These data are mined using complex bioinformatics tools for developing models that could ameliorate diagnosis, prognosis, and prediction accuracy [22–25]. A previous study showed that MR-based radiomics has a potential value in the presurgical prediction of lymph-vascular space invasion in cervical cancer [26]. Another study revealed that radiomics provides a noninvasive approach for analyzing breast cancer subtypes and TN stages [27].

However, there are few reports applying radiomics to assess extrathyroidal extension in PTC, indicating a gap in knowledge. Therefore, MR-based radiomics might provide an accurate approach for extrathyroidal extension prediction in PTC. This work aimed to evaluate whether radiomics applying multiple parametric MRI has the potential to detect extrathyroidal extension in PTC.

## Methods

### Patients

The current retrospective trial assessed consecutive individuals with thyroid nodules firstly identified by US from January 2018 to March 2019. Based on the American College of Radiology Thyroid Imaging, Reporting, and Data System [28], tumor grades were TR3-TR5.

**Fig. 1:**
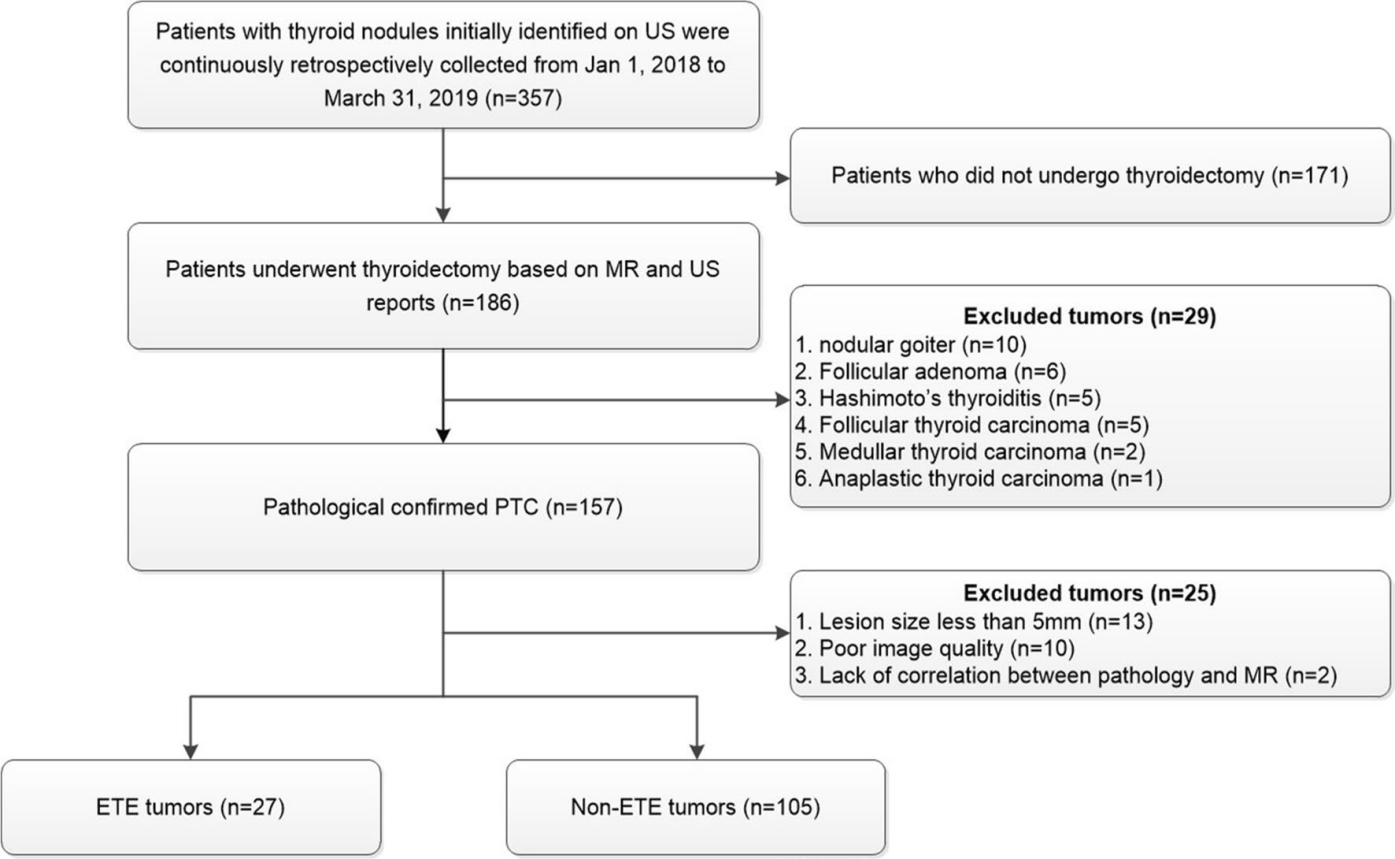
Study flowchart. *US* ultrasound, *PTC* papillary thyroid carcinoma, *ETE* extrathyroidal extension, *MR* magnetic resonance

All patients were examined by multiparametric MRI and subsequently administered thyroid surgery, subtotal or total thyroidectomy, within 1 week following MRI. PTC was pathologically confirmed with surgical specimens. Exclusion criteria were: (1) pathological diagnosis not reflecting PTC; (2) tumor size < 5 mm; (3) no association of pathological data of tumor specimens with MR imaging findings; (4) poor MR quality. Finally, 132 cases were assessed. Figure 1 depicts the patient selection process.

The Institutional Review Board of our Hospital approved this study and waived the requirement for written informed consent due to its retrospective nature.

### MRI acquisition

All patients were scanned on an EXCITE HD 1.5T scanner (GE Healthcare, USA) comprising an 8-channel special neck surface coil, using the same scanning protocol. The applied parameters were as follows: axial T2-weighted (T2WI) fast recovery fast spin-echo with fat suppression with an echo time (TE) of 85 ms, a repetition time (TR) of 1280 ms, a slice thickness of 4–5 mm, a matrix of 288 × 192, spacing of 1 mm, a field of view (FOV) of 18 cm, and a number of excitations (NEX) of 4; DWI with a single-shot echo planar imaging (EPI) sequence, with minimal TE, a TR of 6550 ms, a slice thickness of 4–5 mm, a matrix of 128 × 128, spacing of 0.5 mm, a FOV of 14 cm, and a NEX of 4 (b value, 800 s/mm^2^); contrast-enhanced axial T1WI (CE-T1) with multiphase utilizing a fast-spoiled gradient recalled echo sequence (TE = 1.7 ms, TR = 5.7 ms, matrix = 192 × 256, FOV = 14 cm, and NEX = 1). The Magnevist contrast agent (Bayer Healthcare, USA) was administered by intravenous injection at 3 ml/s (0.2 ml/kg), followed by flushing with 20 ml of normal saline. Scanning was performed at 30, 60, 120, 180, 240 and 300 s after contrast administration, respectively, and images of the six phases were obtained, including breath-holds. Spatial saturation bands were employed for removing signals generated by overlying fat and surrounding tissues.

### Histopathologic analysis

Surgical tumor samples were evaluated and analyzed by an experienced pathologist (> 10 years of related experience). Paraffin-embedding of tumor samples was followed by sectioning and hematoxylin and eosin (H&E) staining. Then, established criteria were utilized by the pathologist for evaluating the extrathyroidal extension (ETE) feature [12]. The patients were then assigned to the non-ETE and ETE groups.

### MRI radiomics

#### Tumor segmentation

ITK-SNAP (http://www.itk-snap.org) was applied for the segmentation of thyroid tumors. Regions of interest (ROIs) were manually drawn on MR images by 2 radiologists (9 and 12 years of related experience, respectively). In case of disagreement, they reached a consensus through additional reading sessions. The ROIs were delineated slice-by-slice to represent the 3D volume of the whole tumor. The largest tumor was selected in each patient and delineated on MR images, which could reduce potential bias of multiple tumors in the same individual and improve the applicability of findings.

#### Radiomics feature extraction

To facilitate imaging analysis, all T2WI, ADC and CE-T1 images were resliced at 4 mm. Radiomic features were automatically extracted with the AK software version 3.2.2 (GE healthcare). A total of 402 features were extracted, including shape, histogram, gray-level run-length matrix (GLRLM), gray-level cooccurrence matrix (GLCM), and gray-level size zone matrix (GLSZM) indexes.

#### Feature selection and model construction

Participants were randomized to the training and test cohorts (ratio, 7:3). To assess interobserver agreement, 30 patients were randomly selected and intraclass correlation coefficients (ICCs) for various features were calculated. According to the 95% confidence intervals (CIs), values below 0.4, from 0.41 to 0.60, from 0.61 to 0.80, and above 0.80 were classified as poor, medium, good, and excellent reliability, respectively. Various features were utilized for further extraction, with ICCs reaching 0.80 [29].

#### Radiomic feature selection

Firstly, the mRMR (maximum correlation minimum redundancy) algorithm was applied in the training group to eliminate redundant and irrelevant features, and 30 features with high correlation with labels, and without redundancy were retained. Then, the least absolute shrinkage and selection operator (LASSO) with ten-fold cross-validation was applied, and the feature subsets was further selected through regularization by optimizing the hyperparameter λ. The coefficients of some candidate features were compressed to zero at the optimal λ, and features with non-zero coefficients were retained for constructing a radiomics signature via a linear combination. Finally, the radiomics score (rad-score) was calculated.

### Model building and validation

The performance of the model in distinguishing the ETE feature of PTC was evaluated and validated by receiver operating characteristic (ROC) curve analysis in the training and test cohorts, respectively. The area under the curve (AUC), sensitivity, specificity, accuracy, and negative and positive predictive values were calculated. In addition, 100 times leave-group-out cross-validation (LGOCV) was carried out to verify the model’s reliability, indicating the results given in the model were not contingent.

## Results

### Patient features

Totally 132 patients aged 45.42 ± 13.99 years (range, 12–77 years) were assessed. Among them, 27 patients (44.89 ± 13.56 years old; age range, 12–73 years) and 105 (45.55 ± 14.10 years old; age range, 22–77 years) were assigned to the ETE and non-ETE groups, respectively, based on pathologic results. ETE patients were divided into those with minimal ETE (n = 15), and gross ETE (n = 12) according to the degree of invasion. Table 1 summarizes the clinical features of PTC cases enrolled in this study. The training cohort included 92 patients, while the testing set had 40 patients.

**Table 1:** Patient features in the ETE and non-ETE groups

	ETE group (n = 27)			Non-ETE group (n = 105)	*P*
	Total	Minimal ETE (n = 15)	Gross ETE (n = 12)		
Age (years)	44.89 ± 13.82	40.13 ± 14.08	50.83 ± 11.38	45.55 ± 14.17	0.828
Diameter (mm)	13.59 ± 6.66	12.24 ± 4.93	15.29 ± 8.26	9.93 ± 4.64	0.001
Sex					
Female	18 (66.7%)	9 (33.3%)	9 (33.3%)	83 (79.0%)	0.363
Male	9 (33.3%)	6 (22.2%)	3 (11.1%)	22 (21.0%)	
Preoperative ultrasound					
Subcapsular location of the tumor	15 (55.6%)			102 (97.1%)	0.000
Extrathyroidal extension	12 (44.4%)	3 (11.1%)	9 (33.3%)	3 (2.9%)	
LN metastasis	13 (48.1%)	5 (18.5%)	8 (29.6%)	27 (25.7%)	0.024
Histological subtype	6 (22.2%)	2 (7.4%)	4 (14.8%)	10 (9.5%)	0.071

### US prediction

Of the 27 patients with ETE, 12 had ETE identified by presurgical US, while the remaining 15 showed no presurgical US evidence of the ETE feature. The sensitivity, specificity and accurate of US were 44.4%, 97.1% and 86.4% in predicting ETE.

### PTC ETE prediction

**Fig. 2:**
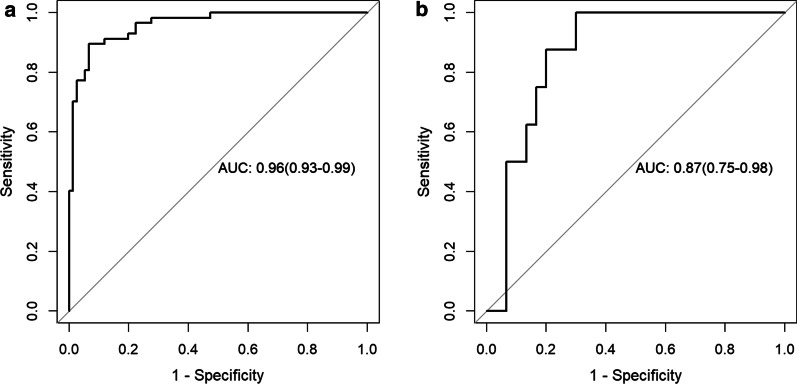
Receiver operating characteristic curves (ROCs) for the radiomics model in predicting ETE and non-ETE tumors in the training (**a**) and test (**b**) cohorts

**Fig. 3:**
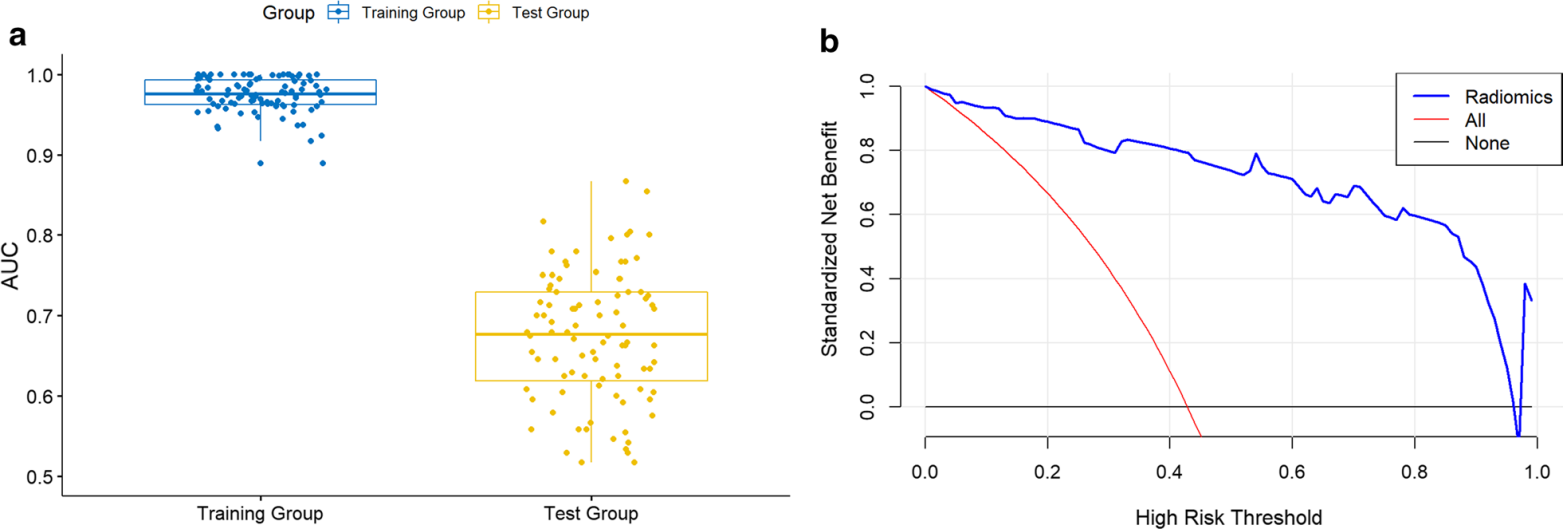
Boxplot of 100 fold LGOCV data (**a**). Decision curve of the radiomics model (**b**) showing that in a threshold range of 0–1, the radiomics model provided a benefit

For predicting ETE and non-ETE masses, 16 top-performing features, including four DWI, seven T2WI, and five CE-T1WI indexes, were finally retained to construct the radiomics signature (Table 2). The proportion of features derived from T2WI was elevated (7/16). There were eight RLM, five CM, two shape and one SZM features. Table 2 shows the coefficients of the selected features. All 16 features showed significant differences between ETE and non-ETE masses (*P* < 0.05). Figure 2 shows ROC curves for the radiomics model in distinguishing ETE from non-ETE masses in the training and test cohorts. The radiomics prediction model yielded AUCs of 0.96 (95% CI 0.93–0.99) and 0.87 (95% CI 0.75–0.98) in the training (Fig. 2a) and test (Fig. 2b) sets, respectively. Figure 3a shows the results of 100 fold LGOCV. The clinical decision curve of the radiomics model is depicted in Fig. 3b. Table 3 shows the radiomics model’s diagnostic performance. Sensitivity, specificity and accuracy were 0.895, 0.934 and 0.917 in the training set, respectively, and 0.750, 0.800 and 0.789 in the test set, respectively. The negative predictive value was 92% in the test group. These results indicated an overall good performance of the prediction model.

**Table 2:** Extracted modeling features predictive of ETE and non-ETE tumors

Feature variable	Coefficient
T1_SmallAreaEmphasis	− 1.974
T2_ClusterProminence_angle45_offset4	1.783
T1_LongRunEmphasis_angle45_offset7	0.51
DWI_ShortRunEmphasis_angle90_offset7	1.184
T2_InverseDifferenceMoment_AllDirection_offset4_SD	− 0.54
T1_RunLengthNonuniformity_AllDirection_offset1_SD	− 0.172
T2_RunLengthNonuniformity_AllDirection_offset7_SD	− 0.064
T2_Elongation	0.589
T1_LongRunHighGreyLevelEmphasis_AllDirection_offset1_SD	− 0.494
T1_SphericalDisproportion	0.497
DWI_LongRunEmphasis_angle90_offset4	0.876
T2_LongRunEmphasis_angle45_offset7	4.905
DWI_GLCMEnergy_angle135_offset4	− 0.13
T2_HaralickCorrelation_angle135_offset7	0.064
DWI_ClusterShade_angle0_offset4	0.04
T2_MinorAxisLength	0.162

**Table 3:** Diagnostic performance of the radiomics model

Group	Accuracy (95%CI)	Sensitivity (95%CI)	Specificity (95%CI)	Positive predictive value (95%CI)	Negative predictive value (95%CI)
Training	0.917 (0.857,0.958)	0.895	0.934	0.911	0.922
Test	0.789 (0.627,0.904)	0.750	0.800	0.500	0.923

## Discussion

The results of this study indicate that radiomics analysis based on multiparametric MRI data has the potential to detect the presence of ETE in PTC. The above findings showed that radiomics features yielded a high AUC in predicting ETE in PTC. According to the 2015 ATA Guidelines [12], thyroid lobectomy or thyroidectomy without prophylactic central neck dissection suffices for treating non-aggressive PTCs. Predicting ETE by radiomics based on MRI data would help clinicians identify individuals likely to benefit from more aggressive initial treatment. Therefore, such tool has an important impact on patient management, especially in cases of low-risk thyroid cancer. This study showed that radiomics based on multi-parameter MRI accurately distinguished ETE from non-ETE in PTC, and these findings are expected to promote the development of a non-invasive method for evaluating ETE in PTC.

Our results demonstrated that US had good specificity and accuracy but low sensitivity in predicting ETE, while MRI radiomics showed better performance. The evaluation by US was relatively subjective and depended on the diagnostic level of the operator. MRI is a noninvasive imaging method without ionizing radiation. It is widely available around the world, with a simple and fast clinical setup. Radiomics provides multiple features extracted from images to quantify tumors, and offers the possibility of revealing differences that the human eye cannot recognize. Radiomic features were obtained from multiparametric MRI comprising T2WI (7/16), ADC (4/16) and CE-T1 (5/16) images. A previous report [3] revealed ADC’s associations with various aggressive features of tumors, and showed that only ETE reached significance. Another study by Hu et al. [19] showed that ADC is effective in assessing aggressiveness using ETE in PTC. Ma et al. [30] found that a radiomics signature utilizing T2WI data could predict the pathological extracapsular extension status in prostate cancer patients. However, no similar study regarding multiparametric MRI-based radiomics for the preoperative assessment of ETE in PTC has been published.

This study extracted multiple radiological features, including shape-based, intensity-related and texture features, which comprehensively reflect the underpinning tumor biology. The LASSO was utilized as the feature selection method. It represents a regression analysis technique performing both regularization and variable selection for enhancing prediction accuracy [31]. The LASSO is considered a promising technique for optimal feature selection, and could combine these radiomic features to generate a radiomic signature [32, 33]. A previous study [34] assessed many feature selection techniques, and LASSO showed an optimal performance.

The above results showed that the MR-based prediction model for differentiating ETE and non-ETE masses achieved high AUC values in both the training (0.96) and test (0.87) groups. It is worth mentioning that each feature in the model had a significant difference between the two groups. Radiomics based on MRI can significantly improve the diagnostic performance. PTC patients could benefit from the entire risk threshold of 0 to 1 according to the decision curve. The radiomics model in this study had more features derived from T2WI (7/16) compared with T1WI and DWI, and the most highly weighted feature was from T2WI. A previous study [35] also showed that features extracted from T2WI achieve a higher prediction performance than those obtained from other sequences, indicating that T2WI may provide more information. The combination of sequences can provide more information than each of them individually [36]. In this study, the proportions of GLRLM (8/16) and GLCM (5/16) features were the largest in the final constructed model. The GLRLM is broadly utilized to extract statistical features [37], whose entries record distributions and relationships of image pixels, which can better reflect regional heterogeneous differences. The GLCM provides a second-order technique to generate texture features for determining associations among combinations of gray levels in image indexes [38], which can reflect internal spatial heterogeneity of the lesions.

The present study had limitations. Firstly, the sample size was modest, which may limit the predictive performance of the model. Indeed, this was an exploratory study and the data were collected from a single institution and lacked validation in external cohorts. Secondly, due to the small sample size of ETE, patients with minimal ETE and gross ETE were categorized in the same group for ETE to enable binary classification. In the future, a large-scale study is warranted to confirm that this method could be used to distinguish ETE from non-ETE in PTC and for further subgroup analysis. Thirdly, the size of the lesions significantly differed between the ETE and non-ETE groups, introducing a potential bias in the interpretation of the radiomics prediction model results. Also, thyroid tumors smaller than 5 mm were not included in this study. Future more advanced MR techniques could improve the detection of smaller tumors. Fourthly, TNM staging and follow-up data were not included for evaluating tumor aggressiveness. PTC generally has a favourable prognosis [11, 39], and our retrospective interval was just one year. Thus, further investigation should be performed.

## Conclusions

Overall, the MRI radiomics approach has the potential to stratify patients according to the ETE status in PTC before surgery, and could help improve therapeutic strategies and patient prognosis.

## Data Availability

The datasets analyzed in this study are available from the corresponding author on request.

## Abbreviations

PTC: Papillary thyroid cancer
ETE: Extrathyroidal extension
MRI: Magnetic resonance imaging
DWI: Diffusion weighted imaging
ADC: Apparent diffusion coefficient
T2WI: T2-weighted imaging
T1WI: T1-weighted imaging
TE: Echo time
TR: Repetition time
FOV: Field of view
ROI: Regions of interest
ICC: Intraclass correlation coefficient
CI: Confidence interval
ROC: Receiver operating characteristic
AUC: Area under the curve
LGOCV: Leave-group-out cross-validation

## References

[1] Guo Z, Ge M, Chu YH, Asioli S, Lloyd RV (2018). Recent advances in the classification of low-grade papillary-like thyroid neoplasms and aggressive papillary thyroid carcinomas: evolution of diagnostic criteria. Adv Anat Pathol.

[2] Song E, Jeon MJ, Oh HS, Han M, Lee YM, Kim TY, Chung KW, Kim WB, Shong YK, Song DE (2018). Do aggressive variants of papillary thyroid carcinoma have worse clinical outcome than classic papillary thyroid carcinoma?. Eur J Endocrinol.

[3] Lu Y, Moreira AL, Hatzoglou V, Stambuk HE, Gonen M, Mazaheri Y, Deasy JO, Shaha AR, Tuttle RM, Shukla-Dave A (2015). Using diffusion-weighted MRI to predict aggressive histological features in papillary thyroid carcinoma: a novel tool for pre-operative risk stratification in thyroid cancer. Thyroid.

[4] Hu A, Clark J, Payne RJ, Eski S, Walfish PG, Freeman JL (2007). Extrathyroidal extension in well-differentiated thyroid cancer: macroscopic vs microscopic as a predictor of outcome. Arch Otolaryngol Head Neck Surg.

[5] Jukkola A, Bloigu R, Ebeling T, Salmela P, Blanco G (2004). Prognostic factors in differentiated thyroid carcinomas and their implications for current staging classifications. Endocr Relat Cancer.

[6] Cushing SL, Palme CE, Audet N, Eski S, Walfish PG, Freeman JL (2004). Prognostic factors in well-differentiated thyroid carcinoma. Laryngoscope.

[7] Shaha AR (2007). TNM classification of thyroid carcinoma. World J Surg.

[8] Cady B, Rossi R (1988). An expanded view of risk-group definition in differentiated thyroid carcinoma. Surgery.

[9] Chung SR, Baek JH, Choi YJ, Sung TY, Song DE, Kim TY, Lee JH (2020). Sonographic assessment of the extent of extrathyroidal extension in thyroid cancer. Korean J Radiol.

[10] Vaisman F, Momesso D, Bulzico DA, Pessoa CH, da Cruz MD, Dias F, Corbo R, Vaisman M, Tuttle RM (2013). Thyroid lobectomy is associated with excellent clinical outcomes in properly selected differentiated thyroid cancer patients with primary tumors greater than 1 cm. J Thyroid Res.

[11] Hay ID (2007). Management of patients with low-risk papillary thyroid carcinoma. Endocr Pract.

[12] Haugen BR (2017). 2015 American thyroid association management guidelines for adult patients with thyroid nodules and differentiated thyroid cancer: what is new and what has changed?. Cancer.

[13] Miller B, Burkey S, Lindberg G, Snyder WH, Nwariaku FE (2004). Prevalence of malignancy within cytologically indeterminate thyroid nodules. Am J Surg.

[14] Liang J, Huang X, Hu H, Liu Y, Zhou Q, Cao Q, Wang W, Liu B, Zheng Y, Li X (2018). Predicting malignancy in thyroid nodules: radiomics score versus 2017 American College of Radiology thyroid imaging, reporting and data system. Thyroid.

[15] Lee CY, Kim SJ, Ko KR, Chung KW, Lee JH (2014). Predictive factors for extrathyroidal extension of papillary thyroid carcinoma based on preoperative sonography. J Ultrasound Med.

[16] Gweon HM, Son EJ, Youk JH, Kim JA, Park CS (2014). Preoperative assessment of extrathyroidal extension of papillary thyroid carcinoma: comparison of 2- and 3-dimensional sonography. J Ultrasound Med.

[17] Cooper DS, Doherty GM, Haugen BR, Kloos RT, Lee SL, Mandel SJ, Mazzaferri EL, McIver B, American Thyroid Association Guidelines Taskforce on Thyroid N, Differentiated Thyroid C (2009). Revised American Thyroid Association management guidelines for patients with thyroid nodules and differentiated thyroid cancer. Thyroid.

[18] Baloch ZW, LiVolsi VA, Asa SL, Rosai J, Merino MJ, Randolph G, Vielh P, DeMay RM, Sidawy MK, Frable WJ (2008). Diagnostic terminology and morphologic criteria for cytologic diagnosis of thyroid lesions: a synopsis of the National Cancer Institute Thyroid Fine-Needle Aspiration State of the Science Conference. Diagn Cytopathol.

[19] Hu S, Zhang H, Wang X, Sun Z, Ge Y, Li J, Dou W. Can diffusion-weighted MR imaging be used as a tool to predict extrathyroidal extension in papillary thyroid carcinoma? Acad Radiol. 2020. 10.1016/j.acra.2020.03.005 32303443

[20] Wang H, Liu K, Ren J, Liu W, Chen Y, Song B (2019). Magnetic resonance imaging characteristics of papillary thyroid carcinoma for the prediction of cervical central compartment lymph node metastasis. J Comput Assist Tomogr.

[21] Schob S, Voigt P, Bure L, Meyer HJ, Wickenhauser C, Behrmann C, Hohn A, Kachel P, Dralle H, Hoffmann KT (2016). Diffusion-weighted imaging using a readout-segmented, multishot EPI sequence at 3 T distinguishes between morphologically differentiated and undifferentiated subtypes of thyroid carcinoma-a preliminary study. Transl Oncol.

[22] Zhou M, Scott J, Chaudhury B, Hall L, Goldgof D, Yeom KW, Iv M, Ou Y, Kalpathy-Cramer J, Napel S (2018). Radiomics in brain tumor: image assessment, quantitative feature descriptors, and machine-learning approaches. AJNR Am J Neuroradiol.

[23] Xu X, Zhang X, Tian Q, Wang H, Cui LB, Li S, Tang X, Li B, Dolz J, Ayed IB (2019). Quantitative identification of nonmuscle-invasive and muscle-invasive bladder carcinomas: a multiparametric MRI radiomics analysis. J Magn Reson Imaging.

[24] Pinker K, Chin J, Melsaether AN, Morris EA, Moy L (2018). Precision medicine and radiogenomics in breast cancer: new approaches toward diagnosis and treatment. Radiology.

[25] Gillies RJ, Kinahan PE, Hricak H (2016). Radiomics: images are more than pictures, they are data. Radiology.

[26] Li Z, Li H, Wang S, Dong D, Yin F, Chen A, Wang S, Zhao G, Fang M, Tian J (2019). MR-based radiomics nomogram of cervical cancer in prediction of the lymph-vascular space invasion preoperatively. J Magn Reson Imaging.

[27] Xie T, Wang Z, Zhao Q, Bai Q, Zhou X, Gu Y, Peng W, Wang H (2019). Machine learning-based analysis of MR multiparametric radiomics for the subtype classification of breast cancer. Front Oncol.

[28] van Griethuysen JJM, Fedorov A, Parmar C, Hosny A, Aucoin N, Narayan V, Beets-Tan RGH, Fillion-Robin JC, Pieper S, Aerts H (2017). Computational radiomics system to decode the radiographic phenotype. Cancer Res.

[29] Koo TK, Li MY (2016). A guideline of selecting and reporting intraclass correlation coefficients for reliability research. J Chiropr Med.

[30] Ma S, Xie H, Wang H, Yang J, Han C, Wang X, Zhang X (2020). Preoperative prediction of extracapsular extension: radiomics signature based on magnetic resonance imaging to stage prostate cancer. Mol Imaging Biol.

[31] Bien J, Taylor J, Tibshirani R (2013). A Lasso for hierarchical interactions. Ann Stat.

[32] Yin P, Mao N, Zhao C, Wu J, Sun C, Chen L, Hong N (2019). Comparison of radiomics machine-learning classifiers and feature selection for differentiation of sacral chordoma and sacral giant cell tumour based on 3D computed tomography features. Eur Radiol.

[33] Ren J, Tian J, Yuan Y, Dong D, Li X, Shi Y, Tao X (2018). Magnetic resonance imaging based radiomics signature for the preoperative discrimination of stage I–II and III–IV head and neck squamous cell carcinoma. Eur J Radiol.

[34] Wang H, Song B, Ye N, Ren J, Sun X, Dai Z, Zhang Y, Chen BT (2020). Machine learning-based multiparametric MRI radiomics for predicting the aggressiveness of papillary thyroid carcinoma. Eur J Radiol.

[35] Yin P, Mao N, Zhao C, Wu J, Chen L, Hong N (2019). A triple-classification radiomics model for the differentiation of primary chordoma, giant cell tumor, and metastatic tumor of sacrum based on T2-weighted and contrast-enhanced T1-weighted MRI. J Magn Reson Imaging.

[36] Wang T, Gao T, Yang J, Yan X, Wang Y, Zhou X, Tian J, Huang L, Zhang M (2019). Preoperative prediction of pelvic lymph nodes metastasis in early-stage cervical cancer using radiomics nomogram developed based on T2-weighted MRI and diffusion-weighted imaging. Eur J Radiol.

[37] Zhang H, Hung CL, Min G, Guo JP, Liu M, Hu X (2019). GPU-accelerated GLRLM algorithm for feature extraction of MRI. Sci Rep.

[38] Arebey M, Hannan MA, Begum RA, Basri H (2012). Solid waste bin level detection using gray level co-occurrence matrix feature extraction approach. J Environ Manag.

[39] Brito JP, Hay ID, Morris JC (2014). Low risk papillary thyroid cancer. BMJ.

